# Long-range and short-range tumor-stroma networks synergistically contribute to tumor-associated epilepsy

**DOI:** 10.18632/oncotarget.7962

**Published:** 2016-03-07

**Authors:** Xiao-Yuan Mao, Tursonjan Tokay, Hong-Hao Zhou, Wei-Lin Jin

**Affiliations:** ^1^ Department of Clinical Pharmacology, Xiangya Hospital, Central South University, Changsha, P. R. China; ^2^ Institute of Clinical Pharmacology, Central South University, Hunan Key Laboratory of Pharmacogenetics, Changsha, P. R. China; ^3^ Center for Life Sciences, National Laboratory Astana, Nazarbayev University, Astana, Republic of Kazakhstan; ^4^ Institute of Nano Biomedicine and Engineering, Department of Instrument Science and Engineering, Key Laboratory for Thin Film and Microfabrication Technology of Ministry of Education, School of Electronic Information and Electronic Engineering, Shanghai Jiao Tong University, Shanghai, P. R. China; ^5^ National Center for Translational Medicine, Shanghai Jiao Tong University, Shanghai, P. R. China

**Keywords:** brain tumor, tumor microenvironment, tumor-associated epilepsy, long-range mode, short-range mode

## Abstract

Epileptic seizures are frequently caused by brain tumors. Traditional anti-epileptic treatments do not acquire satisfactory responses. Preoperative and postoperative seizures seriously influence the quality of life of patients. Thus, tumor-associated epilepsy (TAE) is an important subject of the current research. The delineation of the etiology of epileptogenesis in patients with primary brain tumor may help to find the novel and effective drug targets for treating this disease. In this review, we describe the current status of treatment of TAE. More importantly, we focus on the factors that are involved in the functional connectivity between tumors and stromal cells. We propose that there exist two modes, namely, long-range and short-range modes, which likely trigger neuronal hyperexcitation and subsequent epileptic seizures. The long-range mode is referred to as factors released by tumors including glutamate and GABA, binding to the corresponding receptor on the cellular membrane and causing neuronal hyperactivity, while the short-range mode is considered to involve direct intracellular communication between tumor cells and stromas. Gap junctions and tunneling nanotube network are involved in cellular interconnections. Future investigations focused on those two modes may find a potential novel therapeutic target for treating TAE.

## INTRODUCTION

It has been established that primary brain tumors are one of the most common and lethal cancers worldwide [1–11]. The classification scheme of the World Health Organization (WHO) [12] reports that brain tumors mainly contain these types as follows: glioma, pituitary adenomas, meningiomas, acoustic neurinoma, craniopharyngioma, metastatic tumors.

Tumor microenvironment was shown to be an important contributor to epilepsy in patients with brain tumors and it is estimated that the incidence varies between 30% and 100% depending on the type of tumor [13–15]. For instance, there is a 75% risk of epileptogenesis in the patients with the low grade astrocytoma while glioblastomas carry a 29%-49 risk of epileptic seizures [16], suggesting that malignancies are more epileptogenic [17–21]. Similarly, in 508 Chinese adult patients with low grade gliomas, there are 350 persons (accounting for 68.9%) presented with seizures [22]. Metastatic brain tumor can also induce seizures in about 25% of patients [23]. Nearly 35% of brain tumor patients continue to suffer from spontaneous seizure recurrence, known as tumor-associated epilepsy (TAE), and these patients were often refractory to widely used anti-epileptic drugs such as Valproic acid and Phenytoin [24, 25]. The epileptogenesis and treatment-related problems seriously decreased the quality of life in patients with brain tumor. Up to date, the relationship between epileptogenesis and brain tumor is poorly understood. It was previously hypothesized that epileptogenesis might be associated with tumor invasion as epileptic discharges often appeared from the peri-tumoral region [26–29]. It implies that the intracellular communication exists between tumor cells and stroma cells [30, 31].

## TREATMENT OF TAE

Currently, the traditional anti-epileptic drugs have been employed for the treatment of TAE. These drugs exert anti-epileptic potential via multiple targets. Table 1 displayed the putative mechanism of the common anti-epileptic drugs and the potential therapy for the treatment of TAE. In general, there are two major types of anti-epileptic drugs: first generation drugs (including Valproic acid, Carbamazepine and Phenytoin) and second generation drugs (including Levetiracetam, Lamotrigine and Topiramate) [32, 33]. These drugs have multiple mechanisms of action. For instance, Valproic acid exerts anti-epileptic potential via inhibiting voltage-gated sodium channels and enhancing GABAergic inhibition [34, 35]. The efficacy of traditional anti-epileptic drugs has been studied previously. In a prospective analysis of 26 patients with the primary brain tumor, seizures were significantly decreased by more than 50% in 65% of the patients after treatment with Levetiracetam [27]. Besides, Maschio et al. also found that Levetiracetam treatment reduced seizure frequency by more than 50% in 72% of 19 patients [32] and Newton et al. observed in 90% of 41 patients [36]. Other anti-epileptic drugs studied in patients with a brain tumor are Valproic acid and Topiramate. 55.6% of seizure freedom and 20% of seizure reduction (reduced seizure frequency of more than 50%) were found in a cohort of 47 patients with a brain tumor after add-on and monotherapy with Topiramate [37].

**Table 1:** The proposed targets of common anti-epileptic drugs and the potential therapy for tumor-associated epilepsy

Mechanisms of action	Relevant tumor type	Major potential anti-epileptic drugs
Sodium channels	Glioma	VPA, CBZ, LTG, TPM, PHT, ZNS
GABA	Glioma	VPA, LTG TPM, PB LEV LEV PHT, ZNS
GABA receptors	Glioma	
Potassium channels	Glioma	
SV2A	Glioma	
Enzyme changes	Glioma	
PI3K-mTOR pathway	Glioma	ZNS
AMPA receptors	Gangglioglioma	PB PB
GABA receptors	Gangglioglioma	
Potassium channels	Gangglioglioma	LEV LEV
IL-1β	Gangglioglioma	
PI3K-mTOR pathway	Gangglioglioma	ZNS TPM TPM TPM TPM LEV
AMPA receptors	Gangglioglioma	
GABA receptors Potassium channels Kainate receptors SV2A	Gangglioglioma Gangglioglioma Astrocytoma Glioneuronal tumors	

Note: GG = ganglioglioma; GN = glioneuronal tumours; VPA = valproic acid; TPM = topiramate; CBZ = carbamazepine; PB = Phenobarbital; LTG = lamotrigine; LEV = levetiracetam; PHT = phenytoin; ZNS = zonisamide.

In considering which anti-epileptic drugs to select for treatment of patients with TAE, it is essential for physicians to weigh the benefit and potential harms. As a beneficial aspect, using anti-epileptic drugs could reduce the risk of first seizure or seizure recurrence and improve the quality of life [38]. As a harmful or negative facet, treatment with these drugs may cause related adverse effects, drug-drug interactions and lastly also increase financial burden. Anti-epileptic drugs often trigger a broad range of side effects, such as liver dysfunction and skin rash [39]. Aguiar et al. found that the patients with brain tumor were more vulnerable to the adverse reactions of anti-epileptic drugs than other epileptic patients [40]. During radiotherapy, patients receiving monotherapy with Oxcarbazepine had a higher risk of skin rash such as Stevens-Johnson syndrome [41]. Additionally, phenytoin treatment could also cause 14%-27% of rash in patients with brain tumors [16]. Cognitive deficits were also reported in many patients with brain tumors and might be more common in first generation anti-epileptic drugs such as Phenytoin, Carbamazipine and Valproic acid than the second generation drugs [42, 43]. Indeed, the results of Klein et al. revealed that low-grade glioma patients who used antiepileptic drugs exhibited worse cognitive tests than patients who did not use antiepileptic drugs [44]. Recent investigations have demonstrated that some anti-epileptic drugs generate pharmacokinetic interactions. In addition, Valproic acid was previously found to enhance chemotherapeutic effects in patients with glioblastoma due to its histone deacetylase-inhibiting properties [45].

Glioma patients often undergo chemotherapy during their disease course. Temozolomide was considered as the first-line reagent for treating patients with low-grade and high-grade gliomas [7, 46]. In a group of 30 patients with low-grade gliomas during chemotherapy with temozolomide, 54% of epileptic patients had a reduced seizure frequency and 21% became seizure free, implying that quality of life of these patients was greatly improved after temozolomide treatment [47]. The improvement of TAE after treatment with temozolomide was also verified by Pace et al. [48].

## TUMOR-STROMA CROSS-TALK IN TAE

**Figure 1:**
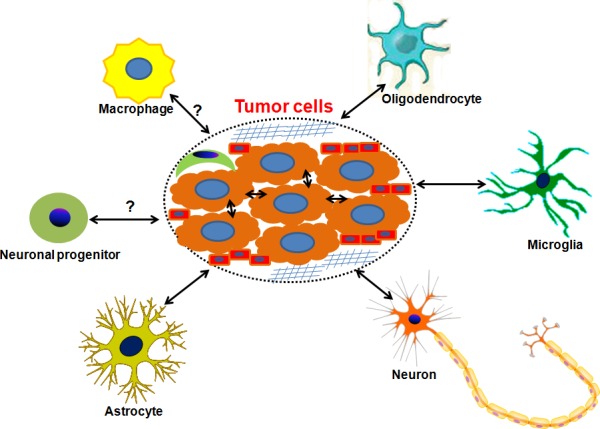
The scheme of brain microenvironment The components in the brain tumor microenvironment are shown in this figure. It is shown that there exists an interaction between brain tumor cell and astrocyte, microglia, oligodendrocyte and macrophage. In the dotted ellipse, brain tumor cells are surrounded by extracellular matrix (
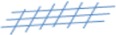
), pericytes (
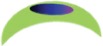
) and endothelial cells (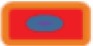).

**Figure 2:**
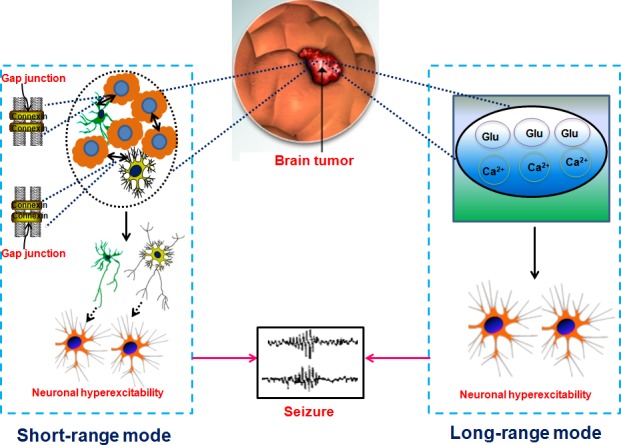
Proposed long-range mode and short-range mode in tumor associated epilepsy We hypothesize that there exists two modes contributing to TAE. One is the long-range mode which indicates that tumor cell released the Glu, Ca2+ and etc, causing neuronal hyperexcitation (as shown in the right part of this figure) and in turn brings out epileptic seizures. The other is the short-range mode which shows that tumor cell interconnects with astrocyte (marked by yellow starriness) or microglia (green starriness-like form) via connectivity such as gap junction (as shown in the left part of this figure) and subsequently activate astrocyte or microglia, finally causing neuronal hyperexcitability and triggering seizures.

The critical roles of tumor microenvironment (TME) on tumorigenesis and tumor progression have been emphasized for many years in multiple types of cancers, including brain tumors [49–54]. Specifically, it was previously illustrated that autocrine factors such as transforming growth factor-α (TGF-α) and heparin-binding epidermal growth factor (HB-EGF) secreted by glioma cells could diffuse through the peri-tumoral stroma and consequently influenced parenchymal cells surrounding the tumor mass [50]. Conversely, normal brain parenchymal cells such as microglia could secret EGF and bind the corresponding receptor EGFR on the glioma cells, providing a permissive microenvironment for malignant glioma progression. Astrocyte-specific deletion of PTEN-targeting microRNAs or blockade of astrocyte exosome secretion suppressed metastasis formation of brain tumor cells [55]. These findings imply that the factors in TME have the capacity to promote tumor progression to some extent. Several types of cells are involved in brain TME, as shown in Figure 1. Typically, they comprise brain tumor cells, astrocytes, microglia, oligodendrocytes, neurons, neuronal progenitors, macrophages, pericytes, endothelial cells and extracellular matrix. In fact, there exists an interaction between brain tumor cell and astrocyte, microglia, oligodendrocyte and macrophage. Recent investigations indicate that glutamate, a well-known excitatory neurotransmitter, is released by glioma cells and causes high extracellular glutamate levels in tumor environment, resulting in neuronal excitotoxicity and the occurrence of TAE [56, 57]. Excessive production of glutamate concentrations in glioma microenvironment is correlated with reduced expression of excitatory amino acid transporter 2 (EAAT2) and increased system xc-cystine/ glutamate transporter (SXC) expression [58]. In fact, a preclinical trial reveals that blockade of SXC by sulfasalazine can remarkably diminish extracellular glutamate content and alleviate epileptic seizures in tumor-bearing mice [57]. These findings imply the central role of the interaction between brain tumor cells and stromas in tumor microenvironment in the etiology of TAE. Intercellular communication is another important facet of tumor-stroma crosstalk. As one of the most important cellular communications, gap junctions were found to exist between tumor cells and stroma cells, finally altering the function of tumor cells [59]. In details, it is shown that functional glioma-glioma gap junctions inhibit glioma invasion while glioma-astrocyte and astrocyte-astrocyte cellular communications promote it in an in vitro transwell invasion assay. What is more important, gap junctions are regarded as a crucial contributor of epileptic seizures [60]. In a genetic model of absence epilepsy, it was observed that epileptic activity was significantly suppressed after treating with carbenoxolone, a gap junction blocker [61]. Collectively, it is summarized that there exists two possible modes that participate in TAE, namely long-range and short-range modes (Figure 2). The long-range mode refers to as factors released by tumors including glutamate, binding to the corresponding receptor on the cellular membrane and causing neuronal hyperactivity, while the short-range mode is considered to involve direct intracellular communication such as gap junctions between tumor cells and stromas.

## THE LONG-RANGE MODE IN TAE: THE GLUTAMATE RELEASE FROM GLIOMA CELLS CAUSED NEURONAL HYPEREXCITABILITY

Glutamate is regarded as the most important excitatory neurotransmitter in the brain. Recently, this excitatory amino acid has been involved in the etiology of glioma. Indeed, the results of the previous microdialysis study revealed the marked elevation of peritumoral glutamate contents in glioma patients (nearly 100 fold higher than levels in uninvolved brain) [62]. This increase in peritumoral glutamate concentration triggers neuronal hyperexcitability, finally leading to tumor-associated seizures [57, 58]. Ye et al. reported that the glutamate release from glioma cells might be ascribed to the activity of SXC, responsible for the cellular synthesis of glutathione (GSH) [56, 63]. Prior work revealed that SXC was highly expressed in 190 glioma patients and the elevated SXC expression was correlated with the occurrence of tumor-related epilepsy [58]. Additionally, the blockade of SXC by sulfasalazine, one U.S. Food and Drug Administration (FDA)-approved SXC inhibitor, was shown to inhibit glutamate release and epileptic seizures [57]. SLC7A11, the catalytic subunit responsible for SXC-induced glutamate release, was also found to be greatly elevated in glioma patients with epileptic activity [64]. And compared with tumors lacking SLC7A11, intracranially implanted SLC7A11-expressing tumors could trigger evident glutamate excitotoxicity and induced seizures [64]. These findings hint that SXC is responsible for releasing glutamate from gliomas and SLC7A11 expression is positively correlated with tumor-associated seizures.

## THE LONG-RANGE MODE IN TAE: LOSS OF GABAERGIC INTERNEURONS AND DEPOLARIZING GABAERGIC RESPONSES

To some extent, it is also important for GABAergic interneurons to maintain the excitation-inhibition balance in the brain [65]. Loss of GABAergic synaptic transmission was previously found to result in neuronal hyperexcitability, finally causing epileptic seizures [66, 67]. L. Campbell et al. disclosed that the marked reduction of peritumoal parvalbumin-positive GABAergic inhibitory interneurons was observed in a mouse glioma model with seizures, accompanied with the deficiency in spontaneous and evoked inhibitory neurotransmission [65]. GABA-induced inhibitory responses are largely activated by A type GABA receptors (GABA_A_Rs) [67]. As is known to all, GABA_A_Rs are ligand-gated chloride-permeable ion channels assembled from a diversity of polypeptide subtypes (α1-α6, β1-β3, γ1-γ3, δ ε, π, θ, ρ1-ρ3) [68]. GABA_A_R-induced fast-hyperpolarizing inhibition relies upon the low intracellular concentration of chloride. The potassium chloride cotransporter 2 (KCC2) is the neuron-specific member of the *SLC12A* family of cation-chloride cotransporters, which mainly extrudes neuronal chloride in adult central nervous system [69, 70]. Previous investigations elucidated that KCC2 was in charge of an inwardly directed electrochemical gradient of chloride and subsequently generated GABA_A_ receptor-induced hyperpolarizing inhibitory responses in adult brain [71, 72]. Indeed, it was previously found that KCC2 could prevent neuronal hyperexcitation in mouse hippocampus [73]. In contrast, KCC2 knockout or deficiency contributed to the development of epilepsy in flies or mice [74]. It is plausible that decreased function of KCC2 can convert GABA to an excitatory neurotransmitter and generate depolarizing GABAergic responses. In a mouse glioma model with peritumoral epilepsy, impaired KCC2 expression was found to induce depolarizing GABA responses due to altered chloride homeostasis [65]. It implies that peritumoral Glu concentration is necessary but not sufficient for TAE and inhibition triggered by GABA is also a central contributor of neuronal excitation.

## THE SHORT-RANGE MODE IN TAE: ALTERATION OF GAP JUNCTION IS OF VITAL IMPORTANCE TO TUMOR-STROMA CROSSTALK

The intracellular communication via gap junctions represents an important pathway to promote cell growth and differentiation [59, 75–77]. Additionally, the intracellular coupling via gap junctions also activates Ca^2+^ signaling in glial cells, which in turn enhances neuronal activity at a distance [78, 79]. Gap junctions are formed by the docking of intracellular channels, each consisting in hexameric arrangements of intrinsic membrane proteins, connexins (CXs) and each six connexins compose one connexon. The expression of CX is cell-type dependent. Neurons (CX43, CX32, CX36), oligodendrocytes (CX32, CX47, CX29), astrocytes (CX43, CX30, CX26) and microglia (CX43, CX36, CX32) express different CXs in the brain [80]. A previous investigation illustrated that CX43 was abundantly expressed in reactive astrocytes surrounding glioma [81], suggesting CX43-induced alteration of gap junctions were involved in the tumor-stroma crosstalk. Besides, previous studies supported the notion that increased glial gap junction coupling was associated with occurrence of epilepsy [82, 83]. Up-regulation of astrocytic CX43 might exacerbate generalized seizures in mesial temporal lobe epilepsy [84]. In addition, blockade of neuronal CX36 channels by Quinine was found to significantly prevent epileptic seizures in experimental animal models [85, 86]. A recent investigation depicted that treatment with a traditional anti-epileptic drug, namely, Valproic acid, remarkably reduced seizure frequency and duration in patients with GBM [87]. As a classical chemotherapeutic agent, Temozolomide was also reported to improve seizure control in glioma patients [84]. And in glioblastoma cells, inhibition of CX43 by a selective blocker called the C-terminal peptide mimetic αCT1, could enhance therapeutic responses in Temozolomide-resistant cancers [88]. It suggests that Temozolomide may alleviate TAE via enhancing gap junctions. Further experimental investigations are essential to verify this speculation.

The direct cellular interconnection can be also performed by tunneling nanotube network (TNN). The TNN was a newly discovered tubular structure between two cells in 2004 [89] and has been found in multiple types of cells, such as rat astrocytes and neurons [90], PC12 cells [91] and mouse macrophage J774 cells [92]. Recently, Zhang et al. found that the tunneling nanotube was formed between rat primary astrocytes and C6 glioma cells [93]. Established tunneling nanotubes between astrocytes and glioma cells significantly inhibited the proliferation of glioma cells. We speculate that the generation of tumor-related seizures may be linked with the tunneling nanotube between glioma cells and human astrocytes.

## PERSPECTIVES

Epilepsy is very common in patients with brain tumors and often not successfully treated after surgical resection. The etiology of TAE is not well understood, but the tumor-induced cellular/molecular alterations which contribute to the changes of surrounding stromas, finally leading to the functional connectivity (Figure 1). We propose two modes are likely to be involved in TAE (Figure 2).

One mode is performed via paracrine mechanisms, namely, secreting neurotransmitters, microvesicles or exosomes by tumor cells and subsequently causing neuronal hyperexcitability and seizures. We consider this mode as a long-range effect on neurons. Indeed, a previous investigation illustrated that glioma-released glutamate had a high risk of seizures in patients [58]. MicroRNA-451/microRNA-21 in extracellular vesicles released from primary human glioblastoma cells were also found to be transferred to microglial and result in the marked reduction of microRNA-451/microRNA-21 target c-Myc mRNA [94].

The other mode refers to the direct cellular communication between tumor cells and adjacent stromas in the microenvironment, which is called short-range mode. In fact, it was reported that brain tumors could interconnect and build a functional network via microtube-associated gap junctions and TNN [89]. We speculate that altered gap junctions and TNN exist between tumor cells and adjacent astrocytes, and these changes may induce tumor-associated epileptic seizures.

In summary, it was for the first time to propose that there exist two major molecular mechanisms, namely, long-range mode and short-range mode, underlying TAE. As mentioned above, glutamate released by tumor cells could alter the biological behaviors of astrocytes and cause neuronal hyperexcitability, finally initiating human epilepsy. However, inhibition of glutamate release generates serious side effects. The other possible mechanisms called short-range mode (cellular interconnection) attract our attention. Further investigations of gap junctions and tunneling nanotube network between tumor and stromas may find a novel effectively therapeutic target for the treating TAE.

## Abbreviations

TAE: tumor-associated epilepsy
TME: tumor microenvironment
TGF-α: transforming growth factor-α
HB-EGF: heparin-binding epidermal growth factor
GBM: glioblastoma multiforme
SXC: system xc- cystine/glutamate transporter
EAAT2: excitatory amino acid transporter 2
GSH: glutathione
FDA: Food and Drug Administration
GABAARs: A type GABA receptors
KCC2: potassium chloride cotransporter 2
CX: connexin
TNN: tunneling nanotube network
